# Characterization of Antibiotic Treatment among Children Aged 0–59 Months Hospitalized for Acute Bacterial Gastroenteritis in Israel

**DOI:** 10.3390/antibiotics13010064

**Published:** 2024-01-08

**Authors:** Muna Omar, Eias Kassem, Roula Abu-Jabal, Basher Mwassi, Dani Cohen, Khitam Muhsen

**Affiliations:** 1 Department of Epidemiology and Preventive Medicine, School of Public Health, Faculty of Medicine, Tel Aviv University, Tel Aviv 6139001, Israel; munaomar@mail.tau.ac.il (M.O.); roulaa@mail.tau.ac.il (R.A.-J.); dancohen@tauex.tau.ac.il (D.C.); 2 Department of Pediatrics, Hillel Yaffe Medical Center, Hadera 3810101, Israel; eiaska@clalit.org.il (E.K.); basher.mwassi@sheba.health.gov.il (B.M.)

**Keywords:** culture-proven bacterial gastroenteritis, hospitalization, antibiotics use, dysentery, children, high-income country

## Abstract

Background: We examined the extent and correlates of appropriate antibiotic use among children hospitalized with bacterial acute gastroenteritis (AGE) in Israel, a high-income country setting. Methods: Data were collected from children aged 0–59 months who participated in active hospital-based surveillance of AGE undertaken during 2007–2015. Bacterial AGE was defined as having a positive stool culture for *Salmonella*, *Shigella*, *Campylobacter*, or dysentery. Appropriate antibiotic use was defined as the administration of ciprofloxacin, azithromycin, or third-generation cephalosporins during hospitalization or at discharge. Results: Overall, 550 children had bacterial AGE; of those, 369 (67.1% [95% CI 63.1–70.9]) received antibiotics, mostly azithromycin (61.8%) and third-generation cephalosporins (37.9%). Appropriate antibiotic treatment was given to 318/550 (57.8% [95% CI 53.7–61.9]). Children aged 0–11 months vs. 24–49 months were more likely to receive appropriate antibiotic treatment (OR = 1.90 [95% CI 1.09–3.33]). Having dysentery (OR = 5.30 [95% CI 3.35–8.39]), performing blood culture (OR = 1.59 [95% CI 1.02–2.48]), and C-reactive protein (CRP) levels (OR = 1.01 [95% CI 1.01–1.02]) were positively associated with receiving appropriate antibiotic treatment. Conclusions: Most children with bacterial AGE received appropriate antibiotic treatment, which correlated with young age, dysentery, CRP level, and performing blood culture, suggesting more severe illness, thus supporting the clinical decisions of physicians.

## 1. Introduction

Acute gastroenteritis (AGE) is a common cause of morbidity and mortality among children [1,2,3], with most diarrheal disease deaths occurring in low-middle-income countries [4,5,6,7]. Viruses are the most common etiologic agents of AGE [2,8]. Rotavirus is the main cause of severe AGE in young children [4,6,9,10], while bacterial enteropathogens account for 10–15% of AGE cases [1,6,10]; most common are *Shigella*, *enterotoxigenic Escherichia coli*, *Campylobacter jejuni*, *Vibrio cholerae*, and *Salmonella* [5,10,11]. 

Given the risk of dehydration, the main treatments for AGE are fluid replacement and appropriate nutrition [1]. Routine antibiotic prescriptions are not recommended for AGE [12], except for more severe bacterial gastroenteritis [13,14], such as that caused by *Shigella*. 

*Shigella* is the main cause of dysentery [13,15,16]. Complications of shigellosis include seizures, hemolytic-uremic syndrome, arthralgia, electrolyte imbalance, and growth faltering in young children [13,16]. Antibiotic treatment is recommended for children with shigellosis, whether it is suspected or culture-proven, to limit *Shigella* infection, increase recovery, and reduce transmission [17]. The recommended first-line antibiotic treatment for shigellosis is ciprofloxacin, and the second-line treatment includes azithromycin, pivmecillinam, and ceftriaxone [14,18,19]. Non-typhoidal *Salmonella* (NTS) is a leading cause of foodborne AGE (salmonellosis) [20,21]. Usually, the disease is self-limiting, but severe illness might develop in immunocompromised individuals, infants, and the elderly [15,21]. About 8% of patients with salmonellosis might develop bacteremia [21], which is prevalent in sub-Saharan Africa [21]. Routine antibiotic treatment for uncomplicated salmonellosis is not recommended since it might increase recurrence and prolong bacterial excretion [18,22]; however, it is recommended for patients at risk of severe disease and bacteremia or extraintestinal infections using third-generation cephalosporins or fluoroquinolone [18,22,23]. AGE caused by *Campylobacter jejuni* [24,25] is mostly self-limited, resolving within 5–7 days without antibiotic treatment [25]. Azithromycin is recommended mainly for dysenteric campylobacteriosis [18,22,26]. 

Several studies examined antibiotic use practices among children with AGE [27,28,29,30,31], showing high rates of antibiotic prescriptions, ranging between 20.0% and 86.9% [27,28,29,31]. While antibiotic use in patients with viral gastroenteritis is unnecessary, antibiotic treatment is needed for patients with some bacterial AGE [18,22] and is prevalent [27,28,29]. The Global Enteric Multicenter Study (GEMS) conducted in sub-Saharan Africa and South Asia (The Gambia, Mali, Mozambique, Kenya, Bangladesh, India, and Pakistan) among 0–59-month-old children showed that *Shigella* was the leading bacterial pathogen driving antibiotic use [28], accounting for 14.9% of all antibiotic prescriptions. In Bangladesh, where approximately half of the dysentery cases occurred, 99.5% of patients with dysentery were given antibiotics, while 73.9% of non-dysenteric moderate-to-severe diarrhea and 57.7% of less-severe diarrhea cases had antibiotic treatment, although they lacked other diagnoses justifying antibiotic administration [28]. The Etiology, Risk Factors and Interactions of Enteric Infections and Malnutrition and the Consequences for Child Health and Development (MAL-ED) study, which was conducted in Bangladesh, Brazil, India, Nepal, Peru, Pakistan, South Africa, and Tanzania [27], showed that *Shigella* and *Campylobacter* attributed to 27.5% and 8.5% of antibiotic use for dysentery, respectively [27]. The most common antibiotic prescriptions were fluoroquinolones, macrolides, and trimethoprim/sulfamethoxazole in these studies [27,28,29,31]. 

Few studies examined antibiotic treatment among children with AGE in high-income countries [30,32,33,34]. A study from South Korea, conducted among children and adolescents less than 18 years of age with acute enterocolitis caused by *Campylobacter*, showed a rate of 36.7% for antibiotic prescriptions (range: 16.7 to 50%), mostly using cephalosporins [32]. A retrospective study conducted in Japan among children and adolescents, aged less than 18 years attending outpatient clinics with infectious diarrhea, showed that 29.6% of the patients had antibiotic prescriptions [30]. A study from Italy compared hospital medical interventions, including antibiotic prescriptions for children hospitalized with AGE, to recommendations by the European Societies of Pediatric Gastroenterology, Hepatology and Nutrition and Pediatric Infectious Diseases guidelines [33] and showed that only 20.6% of the hospitalized patients were managed in full compliance with the guidelines, while 44.7% were managed in partial compliance [33]. It also showed that violation in antibiotic prescription was found in 9.2%, and children with bloody diarrhea and increased inflammatory markers received antibiotic prescriptions more often [33].

Collectively, current evidence suggests that antibiotic treatment is common in children with AGE, some of which might be unnecessary or inappropriate. However, most studies were conducted in low-middle-income countries, where antibiotic prescription patterns and access to antibiotics are likely different compared to high-income countries. Understanding antibiotic prescription patterns in high-income countries is essential to better guide antibiotic stewardship efforts. Accordingly, the aims of the current study were to describe antibiotic prescriptions and factors associated with appropriate antibiotic use among children aged 0–59 months who had been hospitalized with bacterial AGE in Israel, a high-income country. 

## 2. Results

**Figure 1:**
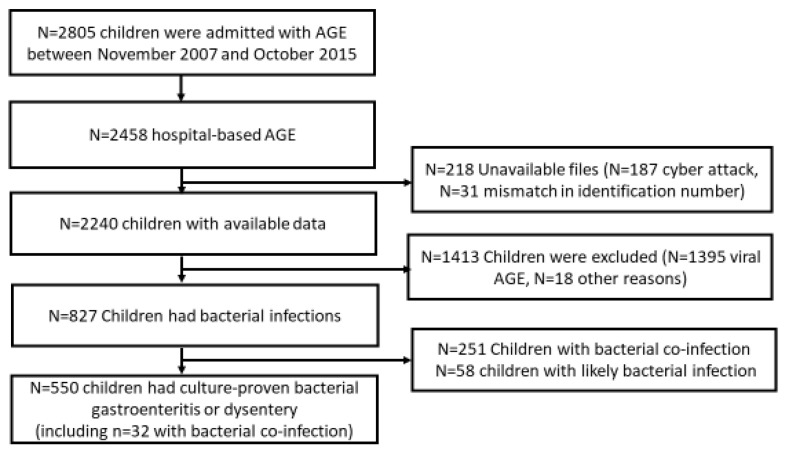
Flow chart of the study Culture-proven bacterial gastroenteritis: positive stool culture for *Salmonella*, *Shigella*, or *Campylobacter.* Likely bacterial infection: blood leukocyte count >15 K/µL, neutrophils level >10 K/µL and C-reactive protein >50 mg/L. Bacterial co-infection: such as pneumonia, urinary tract infection, otitis media, etc. AGE: acute gastroenteritis.

Between November 2007 and October 2015, there were 2805 admissions of children aged 0–59 months with AGE in Hillel Yaffe Medical Center; of those, 2458 (87.6%) were included in the hospital-based AGE surveillance. We had access to data on 2240 (91.1%) of those children, hospitalized between 1 September 2008 and 31 October 2015. Data on 187 children hospitalized from November 2007 to August 2008 (10 months) were missing due to a cyber-attack on the hospital’s computerized system, and records of 31 children could not be found due to a mismatch in identification numbers. Overall, 1413 (63.1%) children were excluded from the study; 1395 since they were classified as having viral gastroenteritis, 18 had justified antibiotic treatment (14 had neonatal fever, 2 had giardiasis, 1 had prophylactic antibiotic treatment before a procedure, and 1 had suspected meningitis), thus leaving in the analysis 827 (36.9%) participants with bacterial infections. Among those, 550 had bacterial AGE based on positive stool culture for *Campylobacter*, *Shigella, Salmonella*, or dysentery; 251 had bacterial co-infection (e.g., UTI, otitis media, pneumonia); and 58 children were classified as likely having bacterial infection (Figure 1).

The mean age of children with bacterial infection (N = 827) was 16.1 months (SD = 14.5), and 51.5% were males. Arab children comprised 57.1% of this sample, and 42.9% were Jewish children. More than 60% of the participants had fever at admission, 42.3% had dysentery, and 70.3% had vomiting. Rotavirus vaccination was administered to 79.1% of children eligible for rotavirus vaccination during the universal vaccination period (birth cohorts 2011–2015) (Table 1).

Stool specimens were obtained from 745 (90.1%) children: 242 (32.5%) were positive for *Campylobacter*, 56 (7.5%) for *Salmonella*, and 65 (8.7%) for *Shigella*. Rotavirus test was performed among 530 (64.1%) children: 23.7% were positive before the introduction of the universal rotavirus vaccination (2008–2010) vs. 7.1% after the introduction of universal rotavirus vaccination (2011–2015). Blood culture was performed in 400 (48.4%) children: 11 (2.7%) of those were positive (*Klebsiella* n = 4, *Streptococcus* spp. n = 3, *Kingella kingae* n = 2, *Staphylococcus aureus* n = 1, *E. coli* n = 1, bacterial entropathogens causing AGE were not detected in blood cultures). Urine culture was performed in 148 (17.9%) children; 37.9% were positive. Chest X-ray imaging was performed in 214 (25.9%) of the children; 55.6% had normal imaging, 7.0% had viral pneumonia, and 32.7% had bacterial pneumonia (Table 2).

Dysentery was found among 350 (63.6%) out of all 550 children; 370 (67.3%) had a positive stool culture for *Campylobacter*, *Shigella,* or *Salmonella*, and 170 (30.9%) children had both dysentery and positive stool culture (of those, 5 had bacterial co-infection). A positive stool culture alone without dysentery was found among 189 (34.4%) children, while 164 (30.0%) had dysentery alone (negative stool culture or stool culture was not performed), and the remaining 27 (4.9%) either had a positive stool culture or dysentery with bacterial co-infection (Table 3). 

Complete blood count and biochemistry tests were performed for most participants. The mean leukocyte level was 14.8 K/µL (SD = 6.7), and the mean neutrophils level was 8.7 K/µL (SD = 6.4). The median C-reactive protein (CRP) level was 32.8 mg/L (IQR 72.3), and, in 38.6% of the children, CRP was above 50 mg/L (Supplementary Table S1).

### 2.1. Antibiotic Treatment among Children Hospitalized with Bacterial AGE

Antibiotic treatment was given to 369 of 550 children with culture-proven bacterial AGE or dysentery, yielding an overall antibiotic use rate of 67.1% (95% CI 63.1–70.9). Among children who were treated with antibiotics, 46 (12.5% [95% CI 9.4–16.1]) received antibiotics before hospital admission, 339 (91.9% [(95% CI 88.7–94.4]) during hospitalizations, and 171 (46.3% [95% CI 41.3–51.5]) had prescriptions on discharge for continuing treatment (Table 4).

### 2.2. Antibiotic Agents

The most commonly used antibiotics were macrolides, namely, azithromycin, given to 61.8% of the participants with bacterial AGE who were treated with antibiotics, followed by third-generation cephalosporins, primarily ceftriaxone, given to 37.9% of the participants, while other antibiotics were less frequently used. Azithromycin was the most common antibiotic treatment given to children before hospital admission (47.8%), during hospitalization (57.5%) (followed by ceftriaxone (40.4%)), and at discharge (83.6%) (Table 4).

Since antibiotic treatment given before hospital admission reflects antibiotic prescription patterns in the community rather than in the hospital setting, further analysis focused on antibiotic treatment during hospitalization or at discharge, which was given to 355/550 children with culture-proven bacterial AGE or dysentery (64.5% [95% CI 60.5–68.5]), with azithromycin and ceftriaxone being the most prescribed antibiotics (59.7% and 39.1%, respectively). Among 242 participants who tested positive for *Campylobacter*, 162 (66.9% [95% CI 60.8–72.7]) were treated with antibiotics, mostly with azithromycin (69.8%) and ceftriaxone (32.7%). Among 65 participants with positive stool culture for *Shigella*, 42 (64.6% [95% CI 52.5–75.5]) were treated with antibiotics: 45.2% received azithromycin, and 42.9% received ceftriaxone. Among 56 participants with positive stool culture for *Salmonella*, 48.2% [95% CI 40.4–61.2]) received antibiotics, mostly azithromycin (55.6%) and ceftriaxone (51.9%). Among 350 children with dysentery, 267 (76.3% [95% CI 71.6–80.5]) received antibiotics, mostly azithromycin (62.2%) and ceftriaxone (37.8%), with similar findings in participants with dysentery alone (Table 5).

Excluded from the analysis, 32 participants who had positive stool culture or dysentery and bacterial co-infection yielded similar results (Supplementary Tables S2 and S3). Of those participants, 31 (96.9% [95% CI 85.5–99.8]) received antibiotic treatment, mostly ceftriaxone (41.9%) and amoxicillin (38.7%) (Supplementary Table S4).

Among the 251 children who had AGE (any) and bacterial co-infection, 236 (94.0% [95% CI 90.6–96.5]), received antibiotic treatment, with amoxicillin being the most prescribed agent (58.5%), followed by ceftriaxone (33.9%) (Supplementary Table S5). 

### 2.3. Correlates of Appropriate Antibiotic Treatment for Bacterial AGE or Dysentery

Appropriate antibiotic treatment with azithromycin or third-generation cephalosporins was given to 318/550 (57.8% [95% CI 53.7–61.9]) participants with culture-proven bacterial AGE or dysentery; 6.7% were treated with other antibiotics, and the remaining (195/550, 35.5%) did not receive antibiotics. The percentage of children aged 0–11 months was higher among participants who received appropriate antibiotic treatment (54.4%) than those who did not (43.1%). Background diseases were slightly less common in the former group (13.2% vs. 20.0%, *p* = 0.041). There were no significant differences between the groups in terms of sex (*p* = 0.803), ethnicity (*p* = 0.135), or residential socioeconomic rank (*p* = 0.371). Dysentery was significantly more common among participants who received appropriate antibiotic treatment vs. those who did not (77.0% vs. 42.6%, *p* < 0.001), while the opposite was found for vomiting (64.8% vs.74.4%, *p* = 0.023). The former group had a higher percentage of children with fever, but the difference was not significant (*p* = 0.097). Performing blood culture and urine culture were significantly more common among participants who received appropriate antibiotics than those who did not, (*p* < 0.001 and *p* =0.004, respectively) but the differences were not significant for performing stool culture (*p* = 0.150) or chest X-ray test (*p* = 0.167). The percentage of children with a positive rotavirus test was lower in the treated group (8.7% vs. 17.3%, *p* = 0.005). The treated group had a higher median CRP level than the untreated group (32.0 mg/dL vs. 17.2 mg/dL, *p* < 0.001), while the mean potassium level (*p* = 0.039) was lower in the former, but the differences were not significant in the mean hemoglobin (*p* = 0.084), leukocytes (*p* = 0.168), and neutrophils (*p* = 0.785) (Table 6).

A multivariable logistic regression model showed that children aged 0–11 months, compared to children aged 24–59 months, were more likely to be prescribed appropriate antibiotic treatment (adjusted OR = 1.90, 95% CI 1.09–3.33). This model showed that children with dysentery had a more than fivefold increased likelihood to receive appropriate antibiotic treatment than children without dysentery (adjusted OR = 5.30, 95% CI 3.35–8.39). Children who had blood culture performed had 1.59 increased odds for appropriate antibiotic treatment than those who did not perform the test (adjusted OR = 1.59, 95% CI 1.02–2.48). This model also showed that, for each 1 mg/L increase in CRP level, the likelihood of administering appropriate antibiotic treatment increased by 1% (adjusted OR = 1.01, 95% CI 1.01–1.02). The associations of vomiting, rotavirus test results, urine culture, and fever with the prescription of appropriate antibiotic treatment were not significant in this model (Table 7). 

## 3. Discussion

In this study, we examined antibiotic use and the correlates of appropriate antibiotic treatment among children aged 0–59 months hospitalized with bacterial AGE in Israel, an example of a high-income country. The main findings of this study were that most (67.1%) children hospitalized with bacterial AGE were treated with antibiotics. The most prescribed antibiotic agents were azithromycin (61.8%) and ceftriaxone (37.9%), resulting in 57.8% of the children with bacterial AGE receiving appropriate antibiotic treatment. The correlates of appropriate antibiotics were younger age, having dysentery, CRP level, and performing blood culture. 

Our findings demonstrate a high percentage of antibiotic use (67.1%) in children with bacterial AGE, reaching 76.3% in children with dysentery. A study from Australia conducted among children aged 15 years or less with AGE showed a high adherence of 97.8% to guidelines for antibiotic use [34]. Another study that involved children aged 6 months to 6 years with AGE showed 70% adherence to the recommended guidelines and that antibiotic treatment was more common among admitted children than non-admitted children [35]. An analysis of the GEMS data showed a high percentage of antibiotic prescriptions: 96.0% for children with moderate-to-severe dysentery aged 0–59 months vs. 71.8% in children with all-cause non-dysenteric moderate-to-severe diarrhea and 54.8% in children with less-severe diarrhea [28]. Findings from the MAL-ED study in children less than 2 years old revealed a small number of diarrheal episodes with dysentery (n = 461, 4.9% episodes), of which 75% received antibiotics [27], in agreement with our findings. The GEMS and MAL-ED studies showed that 14.9% and 11.7% of diarrheal disease antibiotic treatments, respectively, were attributed to *Shigella* [27,28]. Direct comparability of antibiotic prescriptions from our study and GEMS and MAL-ED study might be limited since these studies were conducted in low-middle-income countries, which differ in access to antibiotics and prescription patterns compared to high-income countries [27,28,36,37]. Our and others’ findings [27,28,34,35] suggest that most children with bacterial AGE receive antibiotic treatment in agreement with the recommended guidelines [18,19,23,26,38]. Notably, antibiotic treatment is not recommended routinely for uncomplicated salmonellosis [18,22]. We found that 48.2% of the children with culture-proven *Salmonella* infection received antibiotics, likely empirically, before the result of stool culture was received, given that the disease severity required hospitalization. 

The most used antibiotics in our study were macrolides (61.8%) and third-generation cephalosporins (37.9%), namely, azithromycin and ceftriaxone. Third-generation cephalosporins, including ceftriaxone, are given intravenously or intramuscularly; thus, they were mainly administered during hospitalization, while azithromycin is given orally, and, likely for ease of administering, it was the most prescribed antibiotic before and during hospitalization and at discharge. Ciprofloxacin use was uncommon in our study, likely since it is locally recommended as an alternative antibiotic rather than first-choice antibiotic treatment [38]. Other antibiotic agents, such as penicillin, and first- and second-generation cephalosporins were used at low frequencies, mainly for children who had bacterial co-infection in addition to bacterial AGE or as part of empirical treatment until the workout was completed. 

A study from Japan, a high-income country, involving outpatients under 18 years of age diagnosed with bacterial diarrhea showed that fosfomycin was the most prescribed antibiotic (given to 20.3% of the patients), while macrolides and cephalosporins comprised 3.5% and 4.5% of the treatments, respectively [30]. Fosfomycin is primarily used for urinary tract infections and is not the typical choice for treating diarrheal diseases; therefore, this finding was somewhat surprising. 

The GEMS showed that trimethoprim/sulfamethoxazole was the most prescribed antibiotic agent in the African sites, while quinolones were in the South Asian sites [28]. Unlike other sites, azithromycin was the most used antibiotic in Bangladesh [28]. The most common antibiotics for diarrhea in the MAL-ED study were fluoroquinolone (33.0%) and macrolide (28.0%) [27]. The differences between the leading antibiotic agents that were prescribed in our study and those in the GEMS and MAL-ED studies might reflect variations in access to antibiotics between the various settings, as well as prescription patterns, which might also be affected by economic considerations and antibiotic resistance patterns [27,28,36,37]. 

We found that 12.5% of children received antibiotics before hospital admission. In Israel, only pharmacists dispense antibiotics in outpatient settings and only upon physicians’ prescriptions, like in other high-income countries [39,40]. Antibiotic prescriptions before hospital admission were mainly azithromycin (47.8%), suggesting that the indication was bacterial AGE, while 43.5% received penicillin, suggesting non-specific or other indications for antibiotic treatment.

We found that infants were more likely to receive appropriate antibiotic treatment compared to children aged 24–59 months, in agreement with other studies [28,41]. Likely, infants are at increased risk of severe diarrheal disease, which might explain why infants had higher odds of appropriate antibiotic treatment. 

Dysentery was strongly associated with an increased likelihood of receiving appropriate antibiotic treatment, corroborating findings from the GEMS [28] and a study from Korea [32]. These associations are in line with the guidelines, recommending the administration of antibiotic therapy for children with dysentery [18,22]. Interestingly, in our study, 23.7% of the children with dysentery did not receive antibiotic treatment, suggesting a gap compared to the guidelines. Accordingly, an educational intervention might be warranted to provide optimal treatment for patients with dysentery and reduce the duration of illness. 

We also found a positive association between CRP level and the administration of appropriate antibiotic treatment. CRP is a protein produced by the liver in response to inflammation; it rises in response to infections [42]. A similar positive association between CRP level and early antibiotic treatment in children with diarrhea was reported elsewhere [32,33]. More frequently, antibiotic prescriptions were also prescribed in children with bloody diarrhea and other elevated inflammatory markers [33]. These associations suggest that CRP levels served as an indicator of disease severity that shaped physicians’ decisions toward antibiotic prescription. 

Interestingly, a high proportion, of 48.4%, of the children performed blood culture, which reflects the high availability of diagnostic resources in Israel. We found that performing blood culture was positively associated with an increased likelihood of appropriate antibiotic treatment in children with bacterial AGE. Obolski et al. reported a similar association among children hospitalized for respiratory syncytial virus bronchiolitis but lacking bacterial co-infections [41]. Furthermore, the GEMS demonstrates antibiotic use regarding the severity of the disease; cases with moderate-to-severe diarrhea had more antibiotic prescriptions compared to less severe cases [28]. Performing a blood culture, typically initiated by the attending physician on admission (i.e., before the administration of antibiotic treatment), may suggest a more severe illness and clinical judgment toward bacterial infection. Collectively, our and others’ studies [28,32,33] show that children with bacterial AGE and indicators of more severe illness were more likely to receive appropriate antibiotic treatment. Antibiotic treatment was given before hospital admission for 28 of 240 (11.6%) patients with bacterial AGE who performed blood cultures, which might have affected the growth of bacterial pathogens in blood cultures from these patients.

Our study has some limitations. This was a single-center study, in which antibiotic prescriptions might vary compared to other hospitals. Although the generalizability of the estimated antibiotic use to other hospitals might be limited, they can be generalizable to hospitals with similar profiles. We focused on hospitalized children with bacterial AGE, who likely have more severe illness than in outpatient settings. However, children with severe illness comprise the target population of antibiotic treatment; thus, our study focused on patients who were in most need of antibiotic treatment. The presence of diarrheagenic *Escherichia coli* pathotypes is not routinely tested in clinical settings; therefore, results on the presence of these bacterial enteropathogens were only partially available in our dataset [43] and we cannot exclude the presence of these pathogens in some patients with culture-negative results. Some data were obtained from medical records, which might be incomplete sometimes, due to various documentation practices among physicians and nurses. To overcome this issue, we extracted data from different sources: parental interviews in the original active surveillance study [44,45] and screening of the whole medical record, including admission report, nurses’ follow-up, physicians’ follow-up, laboratory tests, and discharge summary. The data from different sources were cross-checked and verified, yielding a comprehensive and valid dataset, with a low percentage of missing data on most variables. During data collection, the medical center experienced a cyber-attack, which limited our access to some medical records, but this was negligible; as well, some data were missing due to mismatch in identification numbers. If there are differences between physicians or across the years in recording in the medical records, it is likely unrelated to antibiotic treatment. Our definition of bacterial AGE relied in part on having a positive stool culture. The sensitivity of stool culture might be affected by sample quality, bacterial load, and specific techniques used; the test sensitivity had a wide range (60–95%) and specificity (90–99%) [46,47]. It was shown that using PCR increases the detection of *Shigella* in stool specimens obtained from children with diarrhea [46]; thus, we might have missed some of the bacterial AGE. Also, antibiotic administration before stool culture might reduce the accuracy and duration of detection for certain diarrheal pathogens [48]. To increase the chances of including children with bacterial AGE, we included children with dysentery, even in the absence of positive stool culture. 

The dataset did not include information on antibiotic resistance-related bacterial pathogens. A study from Israel showed that *Shigella sonnei* and *Shigella flexneri* isolates had high resistance rates to trimethoprim/sulfamethoxazole and ampicillin (80–100%), while demonstrating very low resistance rates to quinolones and third-generation cephalosporins (1–5%) [49]. This suggests that the majority of *Shigella* isolates were susceptible to the appropriate antibiotic treatment during the study period. Another study reported elevated proportions of resistance to trimethoprim/sulfamethoxazole, tetracycline, and ampicillin among *Salmonella* serotypes [50].

Our study has several strengths. We addressed antibiotic use among hospitalized children with bacterial AGE from a high-income country, thus providing new knowledge on this topic in such settings. Additional strengths include the systematic and comprehensive data collection of demographic, clinical, and laboratory data on a large sample size over a seven-year period. The collected data included information on a wide range of clinical, microbiology, and laboratory results. A stool culture was performed among 90.1% of the children. In addition, handling of stool specimens, including collection, bacterial isolation, incubation, and identification, were processed by standard microbiologic methods and by experienced and qualified staff according to regulatory and accreditation standards. The study hospital serves various population subgroups, including various ethnicities and socioeconomic status levels, increasing our findings’ generalizability to other populations. 

Our study has implications for physicians’ decisions to treat bacterial AGE appropriately by understanding factors that influence antibiotic use in high-income countries. The appropriate antibiotic treatment supports antibiotic stewardship and reduces antimicrobial resistance. 

## 4. Materials and Methods

### 4.1. Study Population and Design

**Figure 2:**
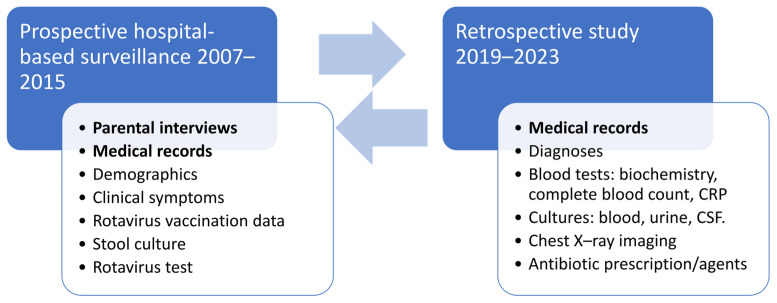
Study design and data collection. Data were collected in two phases. The first phase was a prospective study, hospital-based surveillance conducted during 2007–2015, and included parental interviews, review of medical records, and collection of stool samples, which were tested for culture and rotavirus antigen. The second phase was a retrospective study conducted during 2019–2023, in which complementary data relevant to the current study were obtained retrospectively via full medical chart review. CRP: C-reactive protein; CFS: Cerebral spinal fluid.

This retrospective study was conducted based on active hospital-based surveillance of AGE and rotavirus gastroenteritis (RVGE) undertaken between November 2007 and October 2015 (Figure 2) in the pediatric department of Hillel Yaffe Medical Center in Hadera, Israel [44,45]. The neonatal department and neonatal intensive care unit were not part of this study, as only infants after birth are hospitalized in these wards, while the pediatric department absorbs ill children with acute infectious diseases acquired in the community.

Hillel Yaffe Medical Center is a 500-bed hospital that mainly serves residents in the Hadera sub-district. The study population comprised children aged 0–59 months who received hospitalization services at the hospital’s pediatric department. During the study period, 355,000–410,000 residents lived in the Hadera sub-district; of those, 35,700–39,800 (~10%) were children under 5 years of age. Among children younger than 5 years, 15,300–20,300 (42.9–51.0%) were Jewish, and 19,500–20,400 (54.6–51.3%) were Arab children [51,52]. About 80–90% of the population in the Hadera sub-district receive hospitalization services at Hillel Yaffe Medical Center. 

Access to healthcare services in Israel is universal since all citizens have health care insurance based on the National Health Insurance Law implemented in Israel in 1995 [53]. All citizens are insured in one of Israel’s four health maintenance organizations that provide primary and community-based services. Public hospitals, mostly governmental hospitals, provide in-patient services. The health insurance law covers outpatient and inpatient health services, which provide a basket of services with minimal or no cost to patients at the point of care [53].

In Israel, antibiotics can only be purchased from certified pharmacies with a physician’s prescription. During hospitalization, medications, including antibiotics, are given to patients only based on prescriptions of the attending physician at no cost to patients. 

### 4.2. Data Collection and Definitions 

Data collection was carried out prospectively in the framework of the active surveillance conducted during 2007–2015, and retrospectively in the current study conducted between 2019–2023 to collect complementary data (Figure 2).

AGE was defined as a diarrheal episode in a child 0–59 months of age who was hospitalized in the pediatric department during the study period due to diarrhea (three or more watery stools/24 h). A child hospitalized more than once was considered as having a new episode if he/she was diarrhea-free for at least seven days between hospitalizations. 

Data were collected in the framework of active hospital-based surveillance following the World Health Organization (WHO) generic protocol [54]. Parents of children who were admitted to the hospital for AGE were offered to participate in the study, and those who provided informed consent were enrolled. Information was collected via interviews with the parents, mostly the mothers, and from medical records on demographic factors, including the child’s age on admission (in months), sex (male/female), ethnicity (Arab or Jewish), and residential socioeconomic status (SES)—defined based on the SES rankings of towns of residence as ranked by the Israel Central Bureau of Statistics [55]. The rankings are on a scale of 1 to 10, with 10 being the highest SES. Ranks 1–3 were classified as low SES, while ranks 4–5 and 6–10 were classified as intermediate SES and high SES, respectively. Data were also collected on measured body temperature, number of stools on the most severe day, vomiting, and dysentery (defined as any diarrheal episode in which the loose or watery stool contains visible blood [14]). Fever was defined as body temperature ≥ 38 °C. 

Stool samples were collected from each participant within the first 48 h of hospital admission. Stool specimens were kept at 2–8 °C, transported to the hospital laboratory within a few hours after collection, and tested within 12 h. The presence of *Salmonella*, *Shigella*, and *Campylobacter* in stool was tested by culture. Specimens were tested for rotavirus antigen by immunochromatography (Rotavirus Dipsticks, Hylabs, Rehovot, Israel), following the manufacturer’s instruction.

Following a new ethical approval, new data were retrospectively collected from the hospital medical records of the same children on the following: background diseases, infectious comorbidities, laboratory tests including complete blood count (CBC), CRP, bacteriology (i.e., urine, blood, and cerebrospinal fluid (CSF) cultures), results of chest X-ray imaging, as well as prescriptions for antibiotic treatment and antibiotic class/agents before and during hospitalization and at discharge.

### 4.3. Classification of the Infections 

The current study focused on children with bacterial AGE. Therefore, the first step was to differentiate between viral AGE and bacterial AGE (and sub-categories) and to identify participants with AGE and bacterial co-infections among whom antibiotic treatment might be justified. The classification of infections was undertaken by two investigators: a pharmacist/epidemiologist (MO) and a senior pediatrician (EK), using the entire available clinical and laboratory data. 

The definition of bacterial gastroenteritis combines both clinical symptoms and laboratory confirmation in stool culture [56,57], and it was defined as having a positive stool culture for *Salmonella*, *Shigella*, *Campylobacter***,** or dysentery, even if stool culture was negative or stool culture was not performed. This definition was applied since *Salmonella*, *Shigella*, and *Campylobacter* are the most common causes of bacterial AGE and are routinely tested in clinical settings. Dysentery is usually caused by bacterial enteropathogens such as *Shigella* and *Campylobacter* [56,57]; therefore, it was added to our case definition of bacterial AGE.

Bacterial co-infection was defined as the presence of bacterial extraintestinal infection in addition to AGE. The following bacterial co-infections were considered: urinary tract infection (defined as a positive urine culture with >10^5^ microorganisms per cm^3^), bacteremia defined as positive blood culture—suspected contaminations were excluded (e.g., Coagulase-negative *Staphylococci* and *Viridans Streptococci* group), and otitis media, which included middle ear inflammation, otitis media with effusion, and chronic suppurative otitis media. 

Bacterial pneumonia refers to the interpretation of the chest X-ray imaging, which was undertaken by a physician specialist in radiology, and these summaries were reviewed by a senior pediatrician (EK). In most cases, based on the radiologist’s summaries, we were able to distinguish between a case consistent with bacterial pneumonia or viral pneumonia. In undecided cases, we reviewed the full medical chart, including diagnosis codes, treatment, and physician summaries. Bacterial pneumonia was defined based on a chest radiographic examination showing new or progressive infiltrate, consolidation, cavitation, or pleural effusion following the interpretation of a specialist in radiology, with supporting clinical symptoms and diagnosis. 

Other bacterial co-infections or parasitic infections were also considered (Supplementary Table S6).

Children who did not meet the above-mentioned definitions of bacterial co-infection but had leukocyte levels above 15,000/µL, neutrophils levels above 10,000/µL, and CRP above 50 mg/L were classified as likely to have a bacterial infection.

Children who did not meet all the above-mentioned criteria were classified as having viral AGE and included children with laboratory-confirmed rotavirus gastroenteritis or clinical viral gastroenteritis. 

Appropriate antibiotic use was classified as the administration of ciprofloxacin, azithromycin, or third-generation cephalosporins for children with culture-proven bacterial AGE or dysentery during hospitalization or on discharge. This definition relies on various internal and local guidelines recommending the use of these antibiotics for the treatment of children with bacterial AGE, particularly for those at risk of severe disease, such as young children and those in need of hospitalizations [14,18,19,22,23,26,48,58].

### 4.4. Statistical Methods

The study sample was described using frequencies and percentages for categorical variables, mean and standard deviation (SD), and median and interquartile range (IQR) for continuous variables. The proportion of children who were prescribed antibiotics was calculated and expressed as a percentage with a 95% confidence interval (CI), which was calculated using the binomial-based mid-p method [59]. The analyses were performed for all children with bacterial AGE, and in stratification by sub-categories of bacterial AGE: culture-proven AGE (*Shigella*, *Salmonella*, or *Campylobacter*) or dysentery. The analyses were repeated while excluding children with bacterial AGE and other bacterial co-infections.

To examine factors associated with appropriate antibiotic use, a bivariate analysis was performed using the Mann–Whitney U test for variables with skewed distribution, and the chi-square test and Fisher exact test as appropriate for the categorical variables. Multivariable logistic regression models were used to assess the independent association of each variable with appropriate antibiotic use. Variables were selected to be included in the model based on pre-specified hypotheses that children with markers of more severe diseases (e.g., clinical symptoms, high CRP level, ordering tests by the attending physician, young age) would be more likely to receive antibiotics. Variables with *p* < 0.1 in the bivariate analysis were considered in the multivariable model. Odds ratio (OR) and 95% CI for each variable were obtained from these models. Data were analyzed using IBM SPSS (IBM, Armonk, New York, NY, USA) software version 28 and Winpepi Software [60].

## 5. Conclusions

In conclusion, most children with bacterial AGE or dysentery received appropriate antibiotic treatment in a high-income country setting. Young age, the presence of dysentery, higher levels of CRP, and performing blood cultures were positively associated with appropriate antibiotic use. These indicators suggest severe illness, thus supporting the clinical judgments and decisions made by physicians. Rational use of antibiotics in hospitalized children with bacterial AGE following clinical guidelines is essential for providing optimal care, promoting ideal patient outcomes, and reducing antimicrobial resistance, which might require some educational interventions and training.

## Figures and Tables

**Table 1:** Demographic and clinical characteristics of participants with bacterial infections.

Characteristics	N	%
Overall	827	100.0%
Age (months)		
0–11	416	50.3%
12–23	220	26.6%
24–59	191	23.1%
Sex		
Female	401	48.5%
Male	426	51.5%
Ethnicity		
Jewish	355	42.9%
Arab	472	57.1%
Residential socioeconomic status rank		
1–3 (Low)	397	48.0%
4–5 (Intermediate)	286	34.6%
6–10 (High)	90	10.9%
Missing data	54	6.5%
Years of admissions		
2008 *	31	3.7%
2009	92	11.1%
2010	120	14.5%
2011	74	8.9%
2012	157	19.0%
2013	128	15.5%
2014	154	18.6%
2015	71	8.6%
Fever at admission (≥38 °C)	500	60.5%
Bloody stool (dysentery)	350	42.3%
Vomiting	581	70.3%
Rotavirus vaccination ^¥^		
Birth cohorts not eligible for the universal rotavirus vaccination birth years 2007–2010	45/366	12.3%
Birth cohorts eligible for universal rotavirus vaccination birth years 2011–2015	340/430	79.1%

* Partial data were available for 2008, from 1 September to 31 December. ^¥^ Information on rotavirus vaccination was available for 796 children.

**Table 2:** Microbiological characteristics of children aged 0–59 months hospitalized with bacterial AGE and co-bacterial infections.

	N	%
Overall participants with bacterial infection	827	100.0%
Stool culture		
Stool culture performed	745	90.1%
Stool culture not performed	82	9.9%
Stool culture result (n = 745)		
*Campylobacter*	242	32.5%
*Salmonella*	56	7.5%
*Shigella*	65	8.7%
Mixed infections *	7	0.9%
Any positive stool cultures	370	49.7%
Negative	370	49.7%
Unknown	5	0.7%
Rotavirus test		
Rotavirus test performed	530	64.1%
Rotavirus test not performed	297	35.9%
Rotavirus test result		
Pre-universal rotavirus vaccination period 2008–2010 (n = 190)		
Positive	45	23.7%
Negative	119	62.6%
Unknown	26	13.7%
Universal rotavirus vaccination period 2011–2015 (n = 340)		
Positive	24	7.1%
Negative	312	91.8%
Unknown	4	1.2%
Blood culture		
Blood culture performed	400	48.4%
Blood culture not performed	427	51.6%
Blood culture result (n = 400)		
Positive **	11	2.7%
Negative	340	85.0%
Contamination	8	2.0%
Unknown	41	10.3%
Urine culture		
Urine culture performed	148	17.9%
Urine culture not performed	679	82.1%
Urine culture result (n = 148)		
*E. coli*	43	29.1%
*Klebsiella* (all species) ^¥^	5	3.4%
Other ^§^	8	5.4%
Unknown	17	11.5%
Negative	75	50.6%
Chest X-ray imaging test		
Chest X-ray imaging test performed	214	25.9%
Chest X-ray imaging test not performed	613	74.1%
Chest X-ray test results (n = 214)		
Normal chest X-ray	119	55.6%
Chest X-ray bacterial pneumonia	70	32.7%
Chest X-ray viral pneumonia	15	7.0%
Unknown	10	4.7%

* Mixed infections in stool culture, n = 5 *Campylobacter* and *Salmonella,* n = 2 *Campylobacter* and *Shigella*. ** Positive blood culture: *Klebsiella* n = 4, *Streptococcus* spp. n = 3, *Kingella kingae* n = 2, *Staphylococcus aureus* n = 1, *E. coli* n = 1. ^¥^ *Klebsiella* (all species) in urine culture: *Klebsiella pneumoniae* n *=* 4, *Klebsiella oxytoca* n = 1. ^§^ Other: *Proteus mirabilis* n = 1, *Enterococcus* n = 2; *Citrobacter koseri* n = 1, *Pseudomonas aeruginosa* and *E. coli* n = 1, *E. coli* and *Klebsiella* n = 2, *Proteus mirabilis* and *E. coli* n = 1. AGE = Acute gastroenteritis.

**Table 3:** Classification of infections in participants hospitalized with culture-proven bacterial AGE or dysentery (n = 550) ^¥^.

Category	N	%
Dysentery alone ^§^	164	30.0%
Positive stool culture alone	189	34.4%
Dysentery and positive stool culture	165	30.0%
Dysentery and bacterial co-infections and positive stool culture	5	0.9%
Dysentery and bacterial co-infections *^,§^	16	2.9%
Positive stool culture and bacterial co-infections	11	2.0%
Total	550	100.0%

**^§^** Dysentery without a documented result of positive stool culture. * Bacterial co-infection in addition to AGE. ^¥^ Among 550 children with bacterial AGE, 32 had bacterial co-infections (UTI n = 11, otitis media n = 8, pneumonia n = 6, bacteremia n = 3, cellulitis n = 2, tonsillitis n = 1, mastoiditis n = 1). AGE = Acute gastroenteritis.

**Table 4:** Antibiotic agents prescribed for children hospitalized with culture-proven bacterial AGE or dysentery (n = 550) overall and by timing, relative to hospital admission.

	Antibiotics Treatment (Any)	Before Admission	During Hospitalization	At Discharge
	N (%)	N (%)	N (%)	N (%)
Received antibiotics				
Yes	369/550 (67.1%)	46/369 (12.5%)	339/369 (91.9%)	171/369 (46.3%)
No	181/550 (32.9%)	NA	NA	NA
Antibiotic agents ^§^				
Penicillin				
Ampicillin	7/369 (1.9%)	0/46 (0.0%)	7/339 (2.1%)	0/171 (0.0%)
Amoxicillin	34/369 (9.2%)	17/46 (37.0%)	6/339 (1.8%)	17/171 (9.9%)
Penicillin V	1/369 (0.3%)	1/46 (2.2%)	0/339 (0.0%)	0/171 (0.0%)
Amoxicillin/clavulanic acid	6/369 (1.6%)	2/46 (4.3%)	3/339 (0.9%)	1/171 (0.6%)
Cephalosporins 1st generation				
Cefamezin	3/369 (0.8%)	0/46 (0.0%)	3/339 (0.9%)	0/171 (0.0%)
Cephalexin	11/369 (3.0%)	1/46 (2.2%)	3/339 (0.9%)	8/171 (4.7%)
Cephalosporins 2nd generation				
Cefuroxime	10/369 (2.7%)	0/46 (0.0%)	9/339 (2.7%)	2/171 (1.2%)
Cephalosporins 3rd generation				
Ceftriaxone	140/369 (37.9%)	4/46 (8.7%)	137/339 (40.4%)	0/171 (0.0%)
Ceftazidime	1/369 (0.3%)	0/46 (0.0%)	1/339 (0.3%)	0/171 (0.0%)
Cefotaxime	1/369 (0.3%)	0/46 (0.0%)	1/339 (0.3%)	0/171 (0.0%)
Macrolides				
Azithromycin	228/369 (61.8%)	22/46 (47.8%)	195/339 (57.5%)	143/171 (83.6%)
Other antibiotics				
Gentamicin	5/369 (1.4%)	0/46 (0.0%)	5/339 (1.5%)	0/171 (0.0%)
Metronidazole	6/369 (1.6%)	0/46 (0.0%)	6/339 (1.8%)	2/171 (1.2%)
Trimethoprim/	3/369 (0.8%)	2/46 (4.3%)	0/339 (0.0%)	1/171 (0.6%)
sulfamethoxazole				
Vancomycin	1/369 (0.3%)	0/46 (0.0%)	1/339 (0.3%)	0/171 (0.0%)
Unknown *	15/369 (4.1%)	1/46 (2.2%)	14/339 (4.1%)	0/171 (0.0%)

**^§^** Some received more than one antibiotic agent; therefore, the percentages exceed 100%. * Antibiotic treatment documented without specifying the particular agent used. AGE = Acute gastroenteritis. NA: not applicable. The percentages were calculated among children who received antibiotics.

**Table 5:** Antibiotic treatment during hospitalization or on discharge in children with bacterial AGE or dysentery, overall and by sub-groups.

	Culture-Proven Bacterial AGE or Dysentery	*Campylobacter*	*Shigella*	*Salmonella*	Mixed Infections *	Overall, Positive Stool Culture	Overall Dysentery	Only Dysentery without a Positive Stool Culture
	N (%)	N (%)	N (%)	N (%)	N (%)	N (%)	N (%)	N (%)
Total	550	242	65	56	7	370	350	154
Received antibiotics								
Yes	355/550 (64.5%)	162/242 (66.9%)	42/65 (64.6%)	27/56 (48.2%)	7/7 (100.0%)	238/370 (64.3%)	267/350 (76.3%)	92/154 (59.7%)
No	195/550 (35.5%)	80/242 (33.1%)	23/65 (35.4%)	29/56 (51.8%)	0/7 (0.0%)	132/370 (35.7%)	83/350 (23.7%)	62/154 (40.3%)
Antibiotic agents ^§^								
Penicillin								
Ampicillin	8/355 (2.3%)	3/162 (1.9%)	2/42 (4.8%)	1/27 (3.7%)	0/7 (0.0%)	6/238 (2.5%)	5/267 (1.9%)	2/92 (2.2%)
Amoxicillin	19/355 (5.4%)	9/162 (5.6%)	4/42 (9.5%)	0/27 (0.0%)	1/7 (14.3%)	14/238 (5.9%)	10/267 (3.7%)	1/92 (1.1%)
Amoxicillin/clavulanic acid	4/355 (1.1%)	2/162 (1.2%)	1/42 (2.4%)	0/27 (0.0%)	0/7 (0.0%)	3/238 (1.3%)	3/267 (1.1%)	1/92 (1.1%)
Cephalosporins 1st generation								
Cephalexin	11/355 (3.1%)	6/162 (3.7%)	1/42 (2.4%)	0/27 (0.0%)	0/7 (0.0%)	7/238 (2.9%)	6/267 (2.2%)	2/92 (2.2%)
Cefamezin	3/355 (0.8%)	1/162 (0.6%)	0/42 (0.0%)	0/27 (0.0%)	0/7 (0.0%)	1/238 (0.4%)	2/267 (0.7%)	0/92 (0.0%)
Cephalosporins 2nd generation								
Cefuroxime	10/355 (2.8%)	4/162 (2.5%)	1/42 (2.4%)	1/27 (3.7%)	0/7 (0.0%)	6/238 (2.5%)	2/267 (0.7%)	1/92 (1.1%)
Cephalosporins 3rd generation								
Ceftriaxone	138/355 (39.1%)	53/162 (32.7%)	18/42 (42.9%)	14/27 (51.9%)	4/7 (57.1%)	89/238 (37.4%)	101/267 (37.8%)	31/92 (33.7%)
Ceftazidime	1/355 (0.3%)	0/162 (0.0%)	0/42 (0.0%)	0/27 (0.0%)	0/7 (0.0%)	0/238 (0.0%)	1/267 (0.4%)	1/92 (1.1%)
Cefotaxime	1/355 (0.3%)	1/162 (0.6%)	0/42 (0.0%)	0/27 (0.0%)	0/7 (0.0%)	1/238 (0.4%)	1/267 (0.4%)	0/92 (0.0%)
Macrolides								
Azithromycin	212/355 (59.7%)	113/162 (69.8%)	19/42 (45.2%)	15/27 (55.6%)	2/7 (28.6%)	149/238 (62.6%)	166/267 (62.2%)	58/92 (63.0%)
Other antibiotics								
Gentamicin	5/355 (1.4%)	3/162 (1.9%)	1/42 (2.4%)	0/27 (0.0%)	0/7 (0.0%)	4/238 (1.7%)	1/267 (0.4%)	1/92 (1.1%)
Metronidazole	6/355 (1.7%)	2/162 (1.2%)	0/42 (0.0%)	0/27 (0.0%)	0/7 (0.0%)	1/238 (0.4%)	3/267 (1.1%)	4/92 (4.3%)
Trimethoprim/	1/355 (0.3%)	0/162 (0.0%)	0/42 (0.0%)	1/27 (3.7%)	0/7 (0.0%)	1/238 (0.4%)	1/267 (0.4%)	0/92 (0.0%)
sulfamethoxazole								
Vancomycin	1/355 (0.3%)	1/162 (0.6%)	0/42 (0.0%)	0/27 (0.0%)	0/7 (0.0%)	1/238 (0.4%)	0/267 (0.0%)	0/92 (0.0%)
Unknown ^¥^	14/355 (3.9%)	9/162 (3.7%)	1/42 (2.4%)	0/27 (0.0%)	0/7 (0.0%)	10/238 (4.2%)	8/267 (3.0%)	3/92 (3.3%)

* Mixed infections in stool culture, n = 5 *Campylobacter* and *Salmonella,* n = 2 *Campylobacter* and *Shigella*. **^§^** Some children received more than one antibiotic agent; therefore, the percentages exceed 100%. ^¥^ Antibiotic treatment documented without specifying the particular agent used. AGE = Acute gastroenteritis. Dysentery alone was defined as negative stool culture, or stool culture was not performed. NA: not applicable: the percentages were calculated among children who received antibiotics.

**Table 6:** Demographic and clinical factors associated with appropriate antibiotic use among children hospitalized with bacterial AGE—bivariate analysis.

	Appropriate Antibiotic Treatment ^§^ (N = 318)	No Antibiotic Treatment (N = 195)	*p* Value
Age (months), N (%)			0.045
0–11	173 (54.4%)	84 (43.1%)	
12–23	72 (22.6%)	55 (28.2%)	
24–59	73 (23.0%)	56 (28.7%)	
Sex, males, N (%)	166 (52.2%)	104 (53.3%)	0.803
Ethnicity (Arab vs. Jews), N (%)	210 (66.0%)	116 (59.5%)	0.135
Residential socioeconomic status rank, N (%)			0.371
1–3 (Low)	180 (60.2%)	96 (53.6%)	
4–5 (Intermediate)	90 (30.1%)	63 (35.2%)	
6–10 (High)	29 (9.7%)	20 (11.2%)	
Background diseases, N (%)	42 (13.2%)	39 (20.0%)	0.041
Fever at admission, N (%)	184 (58.0%)	98 (50.5%)	0.097
Vomiting, N (%)	206 (64.8%)	145 (74.4%)	0.023
Dysentery, N (%)	245 (77.0%)	83 (42.6%)	<0.001
Number of stools on the severe day, N (%)			0.563
0–5	104 (32.7%)	59 (30.3%)	
6≤	214 (67.3%)	136 (69.7%)	
Chest-X-ray test performed, N (%)	46 (14.5%)	20 (10.3%)	0.167
Blood culture performed, N (%)	155 (48.7%)	66 (33.8%)	<0.001
Urine culture performed, N (%)	55 (17.3%)	16 (8.2%)	0.004
Rotavirus test results *, N (%)			0.005
Positive	19 (8.7%)	24 (17.3%)	
Negative	193 (88.1%)	104 (74.8%)	
Unknown	7 (3.2%)	11 (7.9%)	
C-reactive protein (mg/L), median (IQR)	32.0 (63.9)	17.2 (38.1)	<0.001
Potassium mEq/L, mean (SD)	4.7 (0.7), N = 280	4.5 (0.6), N = 166	0.039
Hemoglobin (g/dL), mean (SD)	11.5 (1.4), N = 305	11.6 (1.2), N = 186	0.084
Leukocytes (K/µL), mean (SD)	13.4 (5.7), N = 305	13.6 (6.2), N = 186	0.168
Neutrophils (K/µL), mean (SD)	7.7 (6.7), N = 304	7.9 (5.6), N = 185	0.785

**^§^** Receiving azithromycin or cephalosporin third generation. * This analysis included only children who received rotavirus test; 219 children received appropriate antibiotic treatment and 139 children did not receive antibiotics. AGE = Acute gastroenteritis; IQR = Interquartile range; SD: standard deviation.

**Table 7:** Multivariable logistic regression model of factors associated with appropriate antibiotic use among children hospitalized with bacterial AGE or dysentery.

Variable	Unadjusted OR (95% CI)	*p* Value	Adjusted OR (95% CI)	*p* Value
Age (months)	Df = 2	0.082	Df = 2	0.027
0–11	1.54 (1.02–2.33)	0.039	1.90 (1.09–3.33)	0.025
12–23	1.12 (0.69–1.80)	0.651	1.01 (0.56–1.83)	0.972
24–59	Reference group			
Dysentery, yes vs. no	4.53 (3.08–6.66)	<0.001	5.30 (3.35–8.39)	<0.001
Vomiting, yes vs. no	0.63 (0.43–0.94)	0.024	0.65 (0.40–1.07)	0.093
Fever at admission, yes vs. no	1.36 (0.95–1.94)	0.097	1.45 (0.92–2.30)	0.109
C-reactive protein (mg/L), continuous variable	1.01 (1.00–1.01)	<0.001	1.01 (1.01–1.02)	<0.001
Blood culture performed, yes vs. no	1.86 (1.29–2.69)	<0.001	1.59 (1.02–2.48)	0.04
Urine culture performed, yes vs. no	2.34 (1.30–4.21)	0.005	1.12 (0.56–2.24)	0.752
Rotavirus test results	Df = 2	0.048	Df = 2	0.077
Did not perform the test	Reference group			
Positive rotavirus test result	0.45 (0.23–0.89)	0.022	0.66 (0.28–1.54)	0.337
Negative/Unknown test result	0.98 (0.66–1.47)	0.936	1.43 (0.87–2.33)	0.155

N = 461, Nagelkerke R^2^ = 0.269 AGE = Acute gastroenteritis; CI = Confidence interval; Df = Degree of freedom; OR = Odds ratio.

## Data Availability

Data cannot be publicly available given legal and ethical restrictions.

## Supplementary Materials

The following supporting information can be downloaded at: https://www.mdpi.com/article/10.3390/antibiotics13010064/s1. Table S1: Results of complete blood count and biochemistry tests of participants aged 0–59 months hospitalized with bacterial AGE or bacterial co-bacterial infections; Table S2: Antibiotic agents prescribed for children hospitalized with culture-proven bacterial AGE or dysentery without any other bacterial co-infection (n = 518), overall and by timing relative to hospital admission; Table S3: Antibiotic treatment during or after hospitalization in children with culture-proven bacterial AGE, overall and by sub-groups; Table S4: Antibiotic treatment in children with culture-proven bacterial AGE or dysentery with other bacterial infection (N = 32); Table S5: Antibiotic treatment in hospitalized children with AGE and bacterial co-infection; Table S6: Bacterial co-infections with AGE.
