# Supplementary material for: Characterization of Antibiotic Treatment among Children Aged 0–59 Months Hospitalized for Acute Bacterial Gastroenteritis in Israel
Source: Antibiotics (Basel). 2024 Jan 8;13(1):64. doi: 10.3390/antibiotics13010064 (PMC10812600; doi:10.3390/antibiotics13010064)
Supplement: Supplementary file 1 [file antibiotics-13-00064-s001.zip › antibiotics-2770542-supplementary (2).pdf]

Table S1: Results of complete blood count and biochemistry tests of participants aged 0-59 months hospitalized with bacterial AGE or bacterial co-bacterial infections.

Tests	N	Min	Max	Median	IQR	Mean	SD
Hemoglobin (g/dl)	800	7.1	18.9	11.3	1.5	11.4	1.3
Platelets (K/µl)	798	19.0*	872.0	357.0	148	368.8	123.8
Leukocytes (K/µl)	799	2.4	43.8	13.5	8.8	14.8	6.7
Neutrophils (K/µl)	797	0.7	86.1	7.4	7.7	8.7	6.4
Lymphocytes (K/µl)	797	0.4	44.7	4.2	3.4	4.7	3.2
Glucose (mg/dL)	789	32.0	206.0	95.0	23.0	96.9	21.5
Blood urea nitrogen, mg/dL	794	1.6	55.7	9.3	5.9	10.4	5.2
Creatinine (mg/dL)	773	0.04	1.5	0.3	0.1	0.3	0.1
C-reactive protein (mg/L)	796	0.0	369.0	32.8	72.3	56.5	64.3
Sodium mEq/l	759	121.1	189.5	137.0	4.0	137.1	3.7
Potassium mEq/l	729	2.9	7.2	4.6	0.8	4.6	0.6

IQR= Interquartile range; Max= Maximum; Min= Minimum; SD=Standard deviation.
*One child had thrombocytopenia with 19.0 (K/µl) platelets, other values are above> 94 (K/µl).

Table S2: Antibiotic agents prescribed for children hospitalized with culture-proven bacterial AGE or dysentery without any other bacterial co-infection (n=518) overall and by timing relative to hospital admission

	**Antibiotics treatment (any)**	**Before admission**	**During hospitalization**	**At discharge**
	**N (%)**	**N (%)**	**N (%)**	**N (%)**
**Received antibiotic**				
Yes	338/518 (65.3%)	37/338 (10.9%)	310/338 (91.7%)	153/338 (45.3%)
No	180/518 (34.7%)	NA	NA	NA
**Antibiotic agents§**				
**Penicillin**				
Ampicillin	6/338 (1.8%)	0/37 (0.0%)	6/310 (1.9%)	0/153 (0.0%)
Amoxicillin	22/338 (6.5%)	13/37 (35.1%)	4/310 (1.3%)	8/153 (5.2%)
Penicillin V	1/338 (0.3%)	1/37 (2.7%)	0/310 (0.0%)	0/153 (0.0%)
Amoxicillin/clavulanic acid	5/338 (1.5%)	2/37 (5.4%)	2/310 (0.6%)	0/153 (0.0%)
**Cephalosporins 1st generation**				
Cephalexin	7/338 (2.1%)	0/37 (0.0%)	3/310 (1.0%)	5/153 (3.3%)
**Cephalosporins 2nd generation**				
Cefuroxime	4/338 (1.2%)	0/37 (0.0%)	4/310 (1.3%)	0/153 (0.0%)
**Cephalosporins 3rd generation**				
Ceftriaxone	125/338 (37.0%)	3/37 (8.1%)	122/310 (39.4%)	0/153 (0.0%)
Ceftazidime	1/338 (0.3%)	0/37 (0.0%)	1/310 (0.3%)	0/153 (0.0%)
Cefotaxime	1/338 (0.3%)	0/37 (0.0%)	0/310 (0.0%)	0/153 (0.0%)
**Macrolides**				
Azithromycin	221/338 (65.4%)	20/37 (54.1%)	190/310 (61.3%)	140/153 (91.5%)
**Other antibiotics**				
Gentamicin	5/338 (1.5%)	0/37 (0.0%)	5/310 (1.6%)	0/153 (0.0%)
Metronidazole	6/338 (1.8%)	0/37 (0.0%)	6/310 (1.9%)	2/153 (1.3%)
Trimethoprim/sulfamethoxazole	3/338 (0.9%)	2/37 (5.4%)	0/310 (0.0%)	1/153 (0.7%)
Vancomycin	1/338 (0.3%)	0/37 (0.0%)	1/310 (0.3%)	0/153 (0.0%)
**Unknown***	11/338 (3.3%)	0/37 (0.0%)	11/310 (3.5%)	0/153 (0.0%)

§ Some children received more than one antibiotic agent; therefore, the percentages exceed 100%. * Antibiotic treatment documented without specifying the particular agent used. AGE= Acute gastroenteritis. NA: not applicable; percentages calculated among those who received antibiotics

Table S3: Antibiotic treatment during or after hospitalization in children with culture-proven bacterial AGE, overall and by sub-groups

	**Culture-proven bacterial AGE**	***Campylobacter***	***Shigella***	***Salmonella***	**Mixed infections***	**Overall, positive stool culture**	**Overall dysentery**	**Only dysentery without a positive stool**
	**N (%)**	**N (%)**	**N (%)**	**N (%)**	**N (%)**	**N (%)**	**N (%)**	**N (%)**
**Total**	518	230	62	55	7	354	329	154
**Received antibiotics**								
Yes	324/518 (62.5%)	150/230 (65.2%)	40/62 (64.5%)	26/55 (47.3%)	7/7 (100%)	223/354 (63.0%)	246/329 (74.8%)	92/154 (59.7%)
No	194/518 (37.5%)	80/230 (34.8%)	22/62 (35.5%)	29/55 (52.7%)	0/7 (0.0%)	131/354 (37.0%)	83/329 (25.2%)	62/154 (40.3%)
**Antibiotic agents**[§]								
**Penicillin**								
Ampicillin	6/324 (1.9%)	2/150 (1.3%)	1/40 (2.5%)	1/26 (3.8%)	0/7 (0.0%)	4/223 (1.8%)	4/246 (1.6%)	2/92 (2.2%)
Amoxicillin	10/324 (3.1%)	4/150 (2.7%)	2/40 (5.0%)	0/26 (0.0%)	1/7 (14.3%)	7/223 (3.1%)	6/246 (2.4%)	1/92 (1.1%)
Amoxicillin/ clavulanic acid	3/324 (0.9%)	1/150 (0.7%)	1/40 (2.5%)	0/26 (0.0%)	0/7 (0.0%)	2/223 (0.9%)	3/246 (1.2%)	1/92 (1.1%)
**Cephalosporins 1st generation**								
Cephalexin	8/324 (2.5%)	5/150 (3.3%)	1/40 (2.5%)	0/26 (0.0%)	0/7 (0.0%)	6/223 (2.7%)	3/246 (1.2%)	2/92 (2.2%)
**Cephalosporins 2nd generation**								
Cefuroxime	4/324 (1.2%)	2/150 (1.3%)	1/40 (2.5%)	0/26 (0.0%)	0/7 (0.0%)	3/223 (1.3%)	2/246 (0.8%)	1/92 (1.1%)
**Cephalosporins 3rd generation**								
Ceftriaxone	123/324 (38.0%)	48/150 (32.0%)	18/40 (45.0%)	14/26 (53.8%)	4/7 (57.1%)	84/223 (37.7%)	90/246 (36.6%)	31/92 (33.7%)
Ceftazidime	1/324 (0.3%)	0/150 (0.0%)	0/40 (0.0%)	0/26 (0.0%)	0/7 (0.0%)	0/223 (0.0%)	1/246 (0.4%)	1/92 (1.1%)
Cefotaxime	1/324 (0.3%)	1/150 (0.7%)	0/40 (0.0%)	0/26 (0.0%)	0/7 (0.0%)	1/223 (0.4%)	1/246 (0.4%)	0/92 (0.0%)
**Macrolides**								
Azithromycin	207/324 (63.9%)	111/150 (74.0%)	19/40 (47.5%)	15/26 (57.7%)	2/7 (28.6%)	147/223 (65.9%)	162/246 (65.9%)	58/92 (63.0%)
**Other antibiotics**								
Gentamicin	5/324 (1.5%)	3/150 (2.0%)	1/40 (2.5%)	0/26 (0.0%)	0/7 (0.0%)	4/223 (1.8%)	2/246 (0.8%)	1/92 (1.1%)
Metronidazole	6/324 (1.9%)	2/150 (1.3%)	0/40 (0.0%)	0/26 (0.0%)	0/7 (0.0%)	2/223 (0.9%)	5/246 (2.0%)	4/92 (4.3%)
Trimethoprim/ sulfamethoxazole	1/324 (0.3%)	0/150 (0.0%)	0/40 (0.0%)	1/26 (3.8%)	0/7 (0.0%)	1/223 (0.4%)	1/246 (0.4%)	0/92 (0.0%)
Vancomycin	1/324 (0.3%)	1/150 (0.7%)	0/40 (0.0%)	0/26 (0.0%)	0/7 (0.0%)	1/223 (0.4%)	0/246 (0.0%)	0/92 (0.0%)
**Unknown**[¥]	11/324 (3.4%)	7/150 (4.7%)	1/40 (2.5%)	0/26 (0.0%)	0/7 (0.0%)	8/223 (3.6%)	7/246 (2.8%)	3/92 (3.3%)

*Mixed infections in stool culture, n=5 *Campylobacter* and *Salmonella*, n=2 *Campylobacter* and *Shigella*.

[§] Some children received more than one antibiotic agent; therefore, the percentages exceed 100%. [¥] Antibiotic treatment documented without specifying the particular agent used.

AGE= Acute gastroenteritis.

Table S4: Antibiotic treatment in children with culture-proven bacterial AGE or dysentery with other bacterial infection (N=32).

	**Culture proven AGE or dysentery with bacterial co-infection**
**Total**	**N (%)**
	32 (100%)
**Received antibiotics**	
**Yes**	31 (96.9%)
**No**	1 (3.1%)
**Antibiotic agents[§]**	
**Penicillin**	
Ampicillin	1 (3.2%)
Amoxicillin	12 (38.7%)
Amoxicillin/ clavulanic acid	1 (3.2%)
**Cephalosporins 1st generation**	
Cephalexin	3 (9.7%)
Cefamezin	3 (9.7%)
**Cephalosporins 2nd generation**	
Cefuroxime	6 (19.4%)
**Cephalosporins 3rd generation**	
Ceftriaxone	13 (41.9%)
**Macrolides**	
Azithromycin	7 (22.6%)
**Unknown***	4 (12.9%)

[§] Some children received more than one antibiotic agent; therefore, the percentages exceed 100%. * Antibiotic treatment documented without specifying the particular agent used. AGE= acute gastroenteritis.

Table S5: Antibiotic treatment in hospitalized children with AGE and bacterial co-infection

**Total**	**N=251**	**%**
**Received antibiotics**		
Yes	236	94.0%
No	15	6.0%
**Antibiotic agents**[§]		
**Penicillin**		
Ampicillin	13	5.5%
Amoxicillin	138	58.5%
Penicillin V	1	0.4%
Penicillin	1	0.4%
Amoxicillin/ clavulanic acid	36	15.3%
Piperacillin/ tazobactam	1	0.4%
**Cephalosporins 1st generation**		
Cephalexin	19	8.1%
Cefamezin	9	3.8%
**Cephalosporins 2nd generation**		
Cefuroxime	47	19.9%
**Cephalosporins 3rd generation**		
Ceftriaxone	80	33.9%
**Macrolides**		
Azithromycin	21	8.9%
**Other antibiotics**[*]	19	8.1%
**Unknown**[¥]	21	8.9%

[§] Some children received more than one antibiotic agent; therefore, the percentages exceed 100%.
[*]Amikacin (N=1), Clindamycin (N=3), Gentamicin (N=12), Metronidazole (N=1), Trimethoprim/sulfamethoxazole (N=2). [¥] Antibiotic treatment documented without specifying the particular agent used. AGE= acute gastroenteritis.

Table S6: Bacterial co-infections with AGE

	N	%
**Bacterial co-infections with AGE***	251	100.0%
Pneumonia	76	30.3%
Bacteremia	11	4.4%
Urinary tract infection	55	21.9%
Tonsilitis	10	4.0%
Otitis media	93	37.1%
Lymphadenitis	4	1.6%
Impetigo	2	0.8%
Cellulitis	6	2.4%
Osteomyelitis	2	0.8%
Mastoiditis	1	0.4%

*Children with AGE could have one bacterial infection or more therefore, the percentage exceeds 100%.
AGE= Acute gastroenteritis.
